# Bee Pollen Potential to Modulate Ferroptosis: Phytochemical Insights for Age-Related Diseases

**DOI:** 10.3390/antiox14030265

**Published:** 2025-02-25

**Authors:** Rachid Kacemi, Maria G. Campos

**Affiliations:** 1 Observatory of Drug-Herb Interactions, Faculty of Pharmacy, Heath Sciences Campus, University of Coimbra, Azinhaga de Santa Comba, 3000-548 Coimbra, Portugal; 2 Coimbra Chemistry Centre (CQC, FCT Unit 313) (FCTUC), University of Coimbra, Rua Larga, 3000-548 Coimbra, Portugal

**Keywords:** bee pollen, ferroptosis, aging, age-related diseases, cell death, polyphenols, phytochemicals, nutrients

## Abstract

Bee pollen (BP) is one of the richest known natural resources of micronutrients and bioactive phytochemicals. Some captivating bioactivities of BP compounds, although being largely investigated for the latter as individual molecules, remain very scarcely investigated or completely uninvestigated in bee pollen as a whole product. Among the most intriguing of these bioactivities, we identified ferroptosis as a major one. Ferroptosis, a recently discovered form of cell death (connecting oxidative stress and inflammation), is a complex pathophysiological process and one of the most crucial and perplexing events in current challenging human diseases such as cancer, neurodegeneration, and general aging diseases. Many BP compounds were found to intricately modulate ferroptosis depending on the cellular context by inducing this cell death mechanism in malignant cells and preventing it in non-malignant cells. Since research in both fields, i.e., BP and ferroptosis, is still recent, we deemed it necessary to undertake this review to figure out the extent of BP potential in modulating ferroptosis mechanisms. Our research proved that a wide range of BP compounds (polyphenols, phenolamides, carotenoids, vitamins, minerals, and others) substantially modulate diverse ferroptosis mechanisms. Accordingly, these phytochemicals and nutrients showed interesting potential in preclinical studies to lead to ferroptosis-mediated outcomes in important pathophysiological processes, including many aging-related disorders. One of the most paramount challenges that remain to be resolved is to determine how different BP compounds act on ferroptosis in different biological and pathophysiological contexts, either through synergistic or antagonistic behaviors. We hope that our current work constitutes a valuable incentive for future investigations in this promising and very relevant research avenue.

## 1. Introduction

Age-related diseases are becoming one of the most hurdling challenges to global healthcare practitioners and scientists. The United Nations estimates that the worldwide population aged 65+ will reach 1.6 billion in 2050 (nearly double the 2024 number) [1]. Identifying novel targets to prevent and manage age-related diseases is one of the major aims of current biomedical research. In pursuing this aim, natural products have occupied a forefront place in recent years. BP is an unequaled natural pool of micronutrients and bioactive phytochemicals but remains poorly investigated due to its great diversity and the insufficient attention that has been paid to it in biomedical research until the last decade. This cocktail is almost the sole nutritional source of honeybees. Except for sugars (taken from honey) and water, BP provides all other nutrients to honeybee colonies, making the diversity and richness of this matrix crucial for bee and colony health and survival [2]. We have recently published a series of reviews to unveil many novel prospects of BP in human health and age-related diseases [3,4,5]. As a part of this series of untapped potentials, we will, in the current work, review the importance of this bee cocktail as a rich source of ferroptosis modulators.

## 2. Materials and Methods

A bibliographic search was conducted in the main scientific databases specializing in medical and pharmaceutical fields. PubMed, ScienceDirect, Scopus, Web of Science, Cochrane Library, and Google Scholar were included. Different keywords were used, including “ferroptosis “bee pollen” in Google Scholar and “ferroptosis bee pollen” in other databases. Studies about bee pollen as a raw material or a whole extract were absent as of 30 June 2024. As the scientific literature about well-known BP compounds such as polyphenols, carotenoids, and micronutrients was abundant, we decided to establish a list of major BP compounds and search for their relevance to ferroptosis in aging-related pathophysiology. The compound list that we adopted is the same as that which we adopted in our most recent reviews (see [4,5]). To recapitulate, the chosen compounds were apigenin, catechin, chrysin, cyanidin, delphinidin, epicatechin, genistein, hesperidin, hesperetin, isorhamnetin, kaempferol, luteolin, myricetin, naringenin, naringin, pinocembrin, quercetin, and rutin for flavonoids; benzoic, caffeic, chlorogenic, cinnamic, coumaric, dihydroxybenzoic, ellagic, ferulic, gallic, hydroxycinnamic, protocatechuic, rosmarinic, syringic, and vanillic acids for phenolic acids; resveratrol for stilbene derivatives; α-carotene, β-carotene, β-cryptoxanthin, lutein, zeaxanthin, and lycopene for carotenoids; spermidine and its glycosides for phenolamides; betaine and choline for betaines; glucosinolates; and coenzyme Q10. Among BP nutrients, we mainly focused on all vitamins, minerals (copper, iron, selenium, and zinc), and phytosterols.

Firstly, we used search keywords such as “natural”, “phytochemical”, “polyphenol”, “flavonoid”, “nutrient”, “vitamin” coupled with the keyword “ferroptosis” using “AND” Boolean. Then, we searched for publications containing the name of the investigated molecule and the term “ferroptosis”. During the conduction of our work, other terms such as “aging” and “age disease” were included in the search. Some recent articles were consulted to explain ferroptosis mechanisms and their involvement in aging-related pathophysiological processes. The search time interval was limited to the last five years. The initial search returned a great number of articles (around 11,500). A total of 797 papers that met inclusion criteria were selected, classified, and analyzed to conduct our review. Inclusion criteria were mainly the publication in an indexed peer-reviewed journal with a Digital Object Identifier, the reputation of the journal, the publication date, the non-repetitiveness of the paper topic, and the publication in English, Spanish, or French languages. The final number of articles used in the redaction of the current reviews was 250.

## 3. Ferroptosis in Age-Related Diseases

Ferroptosis is a newly discovered (first described in 2012) form of programmed cell death mainly triggered by an intracellular overload of iron and a deficiency in glutathione peroxidase 4 (GPX4); the former resulting in an increased reactive species production through Fenton reactions and the latter resulting from glutathione depletion. These two factors then result in an accumulation of lipid peroxides and subsequent cell death, ultimately driven by membrane lipid peroxidation [6,7]. Other important elementary events characterizing ferroptosis course include cell swelling and plasma membrane condensation; shrinking and increased membrane permeability of the mitochondria; chromatin condensation, albeit the nuclei remain intact; inhibition of the system Xc-(transmembrane cystine/glutamate antiporter), containing the cystine transporter solute carrier family 7 member 11 (SLC7A11, also called xCT, which import extracellular cystine to the cytosol); glutathione (GSH) depletion at least due to the reduced GPX4 activity; and excessive nicotinamide adenine dinucleotide phosphate (NADPH) oxidation [6,7]. Other GSH-independent inhibitors of lipid peroxidation have been discovered and may, therefore, act as regulators of ferroptosis [6,8]. Moreover, ferroptosis can propagate rapidly to other cells, at least in the same tissue, through unclear mechanisms, although lipid peroxide diffusion maybe involved [7,9].

### 3.1. Ferroptosis and Aging Hallmarks

Ferroptosis has been linked to many aging hallmarks that are known as major pathophysiological triggers in age-related diseases (a review of BP potential against aging hallmarks could be retrieved in our recent reviews [4,5]). However, parts of a set of complex and networked interactions involving ferroptosis, other aging-related alterations, and disease triggering and evolution are continuously unveiled. The mechanisms that are involved both in ferroptosis and in this puzzle of interactions remain largely unclear. Ferroptosis has been shown by numerous studies to be tightly linked to inflammation [10], cellular senescence [9], oxidative stress [11], epigenetic regulation [12], mitochondrial dysfunctions [13], autophagy modulation [14], immune response [15], and a wide range of neural alterations [16]. More recent studies also revealed that ferroptosis maybe importantly modulated by novel mechanisms such as proteostasis regulators [17], as well as by external interventions such as microbiota modification [18]. Ferroptosis also promotes aberrant protein aggregation [19]. A recent study in melanoma patients showed that ferroptosis profoundly mediates cellular communication and is involved in the pathogenesis and progression of this cancer type [20], while another study reported that induced NAD+ depletion was involved in promoting ferroptosis in glioma cells [21]. Ferroptosis is also linked in a complex manner to diverse nutrient-sensing pathways, but this link remains to be fully elucidated [22]. In addition, a large number of studies reported that mesenchymal stem cell administration suppressed ferroptosis in diverse tissues [23]. A recent study also demonstrated that telomere alteration by inhibiting one of its capping protective proteins (telomeric repeat-binding factor 2) led to ferroptosis in gastric cancer cells [24]. The complex interplay between ferroptosis mechanisms and all these aging hallmarks makes this form of cell death a very crucial target in tackling many age-related diseases.

**Figure 1:**
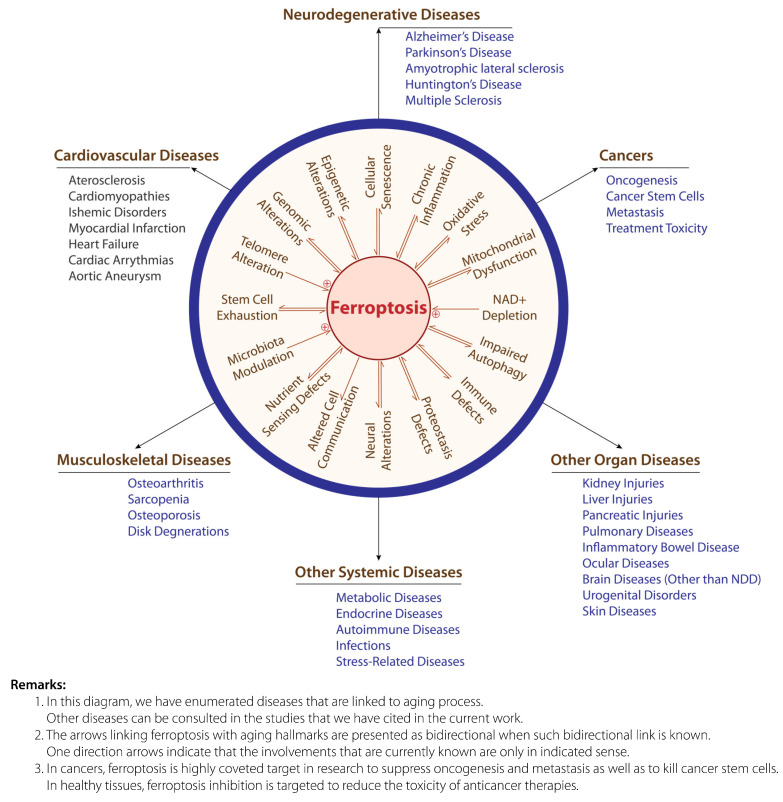
Aging hallmarks and age-related diseases linked to ferroptosis. Adapted from [5,9,10,11,12,13,14,15,16,17,18,19,20,21,22,23,24,25,26,30,31,33,34].

A summary of ferroptosis connection with aging hallmarks and human diseases is presented in Figure 1.

### 3.2. Ferroptosis in Major Age-Related Diseases

Ferroptosis has been specially found to crucially participate in neurodegeneration pathogenesis and to inhibit malignant cell proliferation, hence being coveted as a therapeutic target both through its induction in cancer cells and its inhibition in neurodegeneration [25,26]. In addition to involvement in nerve cell death, ferroptosis has been found to be implicated, through multiple mechanisms, in inducing structural, biochemical, and functional alterations of blood–brain barrier (BBB) [27]. Such alterations are evidently known to be a major hallmark of neurodegenerative diseases (NDD) and diverse central nervous system (CNS) injuries. It is, however, important to note that, despite the substantial amount of preclinical and clinical evidence, how ferroptosis is involved in neurodegeneration etiology and progression, as well as how therapeutic targeting may be approached to result in successful clinical outcomes, remains poorly understood. Similar hindrances may be noted in cancer treatment, although possibly at lower levels of complexity due to a better understanding of oncological mechanisms. Although the number of drugs targeting ferroptosis remains limited due to the novelty of the topic, this targeting is an area of very active research. In cancers, natural products may beneficially modulate ferroptosis through diverse mechanisms. They may induce it in ferroptosis-sensitive tumors or reverse drug resistance and enhance malignant cell sensitivity to therapies, in addition to the fact that some phytocompounds have been found to induce ferroptosis in malignant cells and to inhibit it in normal cells with underlying mechanisms remaining to be elucidated [28]. In cardiovascular diseases, ferroptosis is also a recent mechanism and much pursued target in recent years. Ferroptosis is a risk factor for many cardiovascular diseases (e.g., atherosclerosis, myocardial infarction, cardiac arrhythmia, and other myocardial and ischemic disorders) that promotes diverse changes, such as vascular and muscular cell alterations, oxidative stress, inflammation, hypercoagulation, immune defects, and ion channel dysfunctions that trigger diseases; it also interferes with other forms of cell death in the cardiovascular system [29]. A large number of studies verified the deep involvement of ferroptosis in a wide spectrum of human diseases (recent general reviews can be found in [25,30,31,32]).

## 4. Bee Pollen as a Source of Ferroptosis Modulators

To our current knowledge, studies directly focusing on the bioactivity of BP as a whole product on ferroptosis are still absent. Some mechanisms that are directly related to ferroptosis were, however, reported for some BPs. Indeed, experimental studies reported that *Fagopyrum esculentum*, *Schisandra chinensis*, and other multifloral BPs reduced the levels of lipid peroxidation assessed by its byproduct malondialdehyde (MDA), while *Schisandra chinensis* BP significantly increased glutathione level [35]. MDA is also used as a ferroptosis biomarker [36,37]. Moreover, many BP compounds have been reported to intricately inhibit or induce ferroptosis in preclinical experiments.

**Figure 2:**
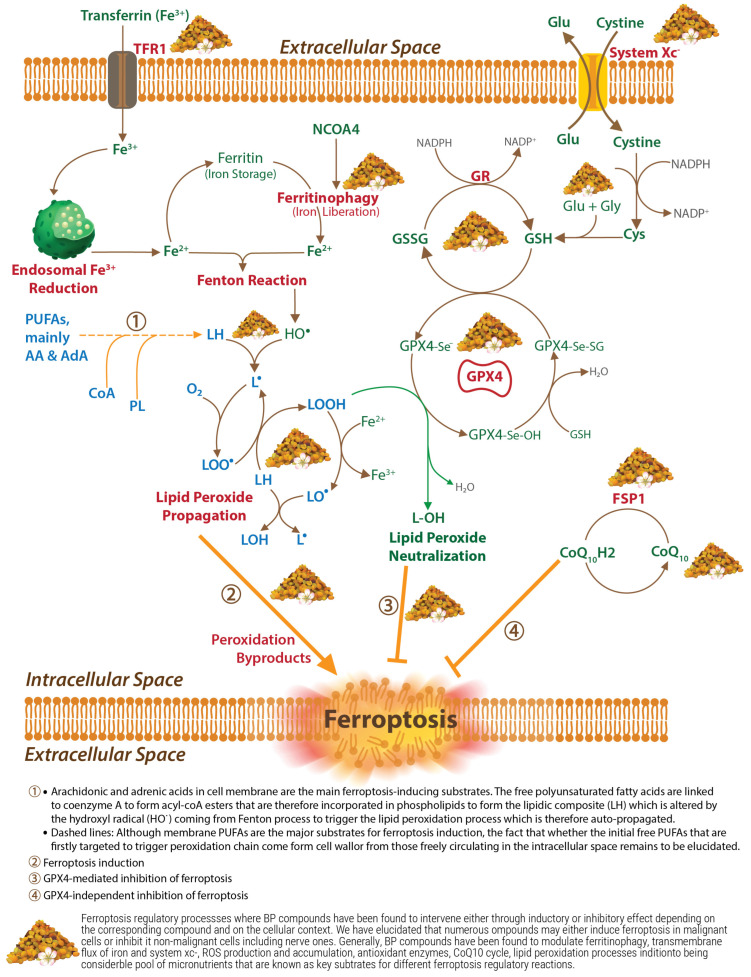
Mechanisms of ferroptosis modulation by key BP compounds. Cys: Cysteine; Gly: glycine; Glu: glutamate; GPX4: glutathione peroxidase 4; GR: glutathione reductase; GSH: glutathione (reduced form); GSSG: glutathione disulfide (oxidized glutathione); L-OH: reduced lipids (alcohols); L-OOH: oxidized lipids (peroxides); NCOA4: nuclear receptor coactivator 4.

A wide range of polyphenols, including many ubiquitous BP compounds, have been shown to exert potent ferroptosis inhibitory effects. For good recent reviews, see [38,39]. It is also important to note that most studies investigating phenolic compounds in ferroptosis as it relates to aging-related diseases were published in the last three–five years, depending on the molecules, with a great part having just been published in the last few months. Data are still raw, and despite the promising results that emanate from preclinical evidence, our understanding of these effects remains elementary, and further research works are needed to end up in beneficial clinical outcomes. Phenolamides are also recently being reported as very abundant and ubiquitous compounds of BP, but studies on their ferroptosis modulatory potential are still lacking, apart from some examples for spermidine that we will see. Ferroptosis regulation activity has also been widely investigated for other ubiquitous BP phytochemicals and nutrients. In the current section, we will review cumulated evidence about all these BP compounds and at the end of the text, in Figure 2, all the following information will be summarized.

### 4.1. Flavonoids

Our review permitted us to identify flavonoids among the most widely studied BP compounds in ferroptosis modulation. Interestingly, these widespread compounds are endowed with intricate and important potential for such modulation in age-related pathophysiological contexts.

#### 4.1.1. Flavanols

Quercetin was found to induce ferroptosis in human hepatocarcinoma and breast and colorectal cancer cell lines and to dramatically reduce the viability of all these cells [40]. It is very important to note that a p53 inhibitor, as well as p53 gene knockout, did not affect this effect. Another important point is that quercetin markedly enhanced nuclear translocation of the core regulator of lysosomal autophagy, namely the transcription factor e-box (TFEB), which was then involved in inducing lysosomal ferritinophagy. Obviously, an important increase in intracellular iron load and lipid peroxidation was reported. Another recent study also reported that quercetin inhibited breast cancer cell proliferation through inducing ferroptosis [41]. The authors of this study also unveiled TFEB-mediated lysosomal ferritinophagy as an underlying molecular mechanism of the observed effects and reported a concomitant increase in intracellular levels of iron and MDA. A network pharmacology analysis has also referred to the marked potential of quercetin, compared to other molecules, to protect against intracellular iron overload and reactive oxygen species (ROS) production and to inhibit ferroptosis, a potential which was further confirmed in a fibroblast-like murine cell line [42]. The potential of quercetin appears, therefore, important in inducing ferroptosis in cancers, especially when it is not possible to reach palpable outcomes by targeting p53-mediated apoptosis. Interestingly, high p53 levels may also enhance ferroptosis [43], which makes quercetin also interesting in p53-rich cancers. Note that p53 involvement in ferroptosis modulation varies according to the cell type and state. In a few examples, such as colorectal cancer and fibrosarcoma, p53 may trigger some ferroptosis-antagonizing mechanisms [44].

On the other hand, to illustrate quercetin-mediated modulation of ferroptosis in NDD, many studies have reported interesting inhibitory effects of this phenolic in non-malignant cells. Quercetin rescued dopaminergic neurons against ferroptosis that was experimentally induced by diverse agents in vitro and in vivo [45]. This study unveiled many underlying mechanisms and experimental outcomes, including mitochondrial structure and function improvements, nuclear factor erythroid 2-related factor 2 (Nrf2) and GPX4 upregulation, suppression of behavioral impairments and neuronal loss, and mitigation of ROS genesis. In rat models of subarachnoid hemorrhage, quercetin suppressed early brain injury by inhibiting ferroptosis in cortical neurons. This was also manifested by rescuing BBB and neuronal function and was biochemically accompanied by a reduction in intracellular iron load and lipid peroxidation and an increase in glutathione and GPX4, along with correcting other alterations in iron transporters and mitochondria [46].

Quercetin has been reported to suppress ferroptosis concomitantly with decreasing iron, reactive species, and lipid peroxidation levels and increasing GPX4 levels in cell lines used as models of epilepsy and spinal cord injury [47]. Similar protective results were also reported in vitro and in vivo against experimental injuries of the kidney (diabetic nephropathy and acute kidney injury models), liver (non-alcoholic fatty liver disease and induced hepatotoxicity models), and bone marrow (bone marrow-derived mesenchymal stem cells, with a likely mitigating role against osteoporosis) [47]. In animal models of sepsis-induced cardiomyopathy, quercetin also acted as a ferroptosis suppressor and ameliorated this cardiac alteration [48]. Quercetin also suppressed ferroptosis in pancreatic β-cell islets of animal models of type 2 diabetes mellitus and mediated a normalization of glucose tolerance and insulin sensitivity in addition to correct glutathione and GPX4 levels and mitochondrial alterations [49].

Bio-informatics-based technics are frequently used to decipher potential candidates, either for the inhibition or induction of ferroptosis, due to the newness of this research topic. A recent screening from a group of 363 molecules (chosen by a machine learning model) identified quercetin, trans-resveratrol, and tocopherol as three of the top five ingredients that bind well to identified ferroptosis-associated hub genes in Parkinson’s disease (PD) [50]. Quercetin was the second most potently binding molecule, after 17-β-estradiol, to all chosen genes (there were six in total).

Nuclear receptor coactivator 4 (NCOA4) is a cytosolic cargo receptor of ferritin that orchestrates its degradation by ferritinophagy after lysosome binding [51,52]. NCOA4 is induced by autophagy processes at least via autophagy-related genes 5 and 7, while NCOA4 knockdown was found to decrease intracellular levels of ferrous ion (Fe^2+^) and lipid peroxidation and suppress ferroptosis [53]. Ferritinophagy is, thus, regarded as having a crosstalk channel with ferroptosis [54], in addition to many other selective autophagy pathways that maintain such crosstalk [55,56]. Consequently, we could envisage that autophagy and/or NCOA4 modulation could result in modulating ferroptosis. Modulation of the crosstalk between ferroptosis and autophagy has recently become a highly coveted topic in cancer research, and promising or sometimes divergent results have been reported [57,58]. In neurodegeneration, studies are still more scarce concerning this crosstalk. A very recent study showed that autophagy induction resulted in suppressing ferroptosis and consequently alleviating cognitive decline in murine models [59]. Inhibiting autophagy-dependent ferroptosis was verified for quercetin in experimentally injured livers of animal models, and marked downregulation of NCOA4, i.e., of ferritinophagy, was revealed as an underlying mechanism [60]. In a ferroptosis model of PD induced in a dopaminergic primary neuron cell line, quercetin has also been reported to suppress ferroptosis with NCOA4 inhibition as an underlying mechanism, at least partly [61]. These three studies are very recent (all have just been published this year), but they open an untapped research avenue to modulate ferroptosis in neurodegeneration (inhibition) and cancer (induction) through autophagy. The latter is relatively well studied as a target of many natural compounds, including many examples of BP that we have previously seen [4].

A very interesting observation to note is that quercetin, in healthy cells, has been reported to selectively inhibit ferroptosis without altering cell viability after experimentally induced apoptosis, necrosis, or autophagy in rat epithelial and human tubular kidney cell lines [62]. The understanding of underlying mechanisms in such an observation may pave the way to selectivity prospection in phenolic and natural compounds in general and to make even combined therapies more profitable. Note that the selective effect of quercetin was observed in both rat and human cell lines. The quest for this so-desired aim among the scientific and clinical communities may also be uplifted by other observations which are recently reported after the introduction of senolytic molecules in biomedical research. Indeed, as it was described for a few natural compounds, quercetin was repeatedly reported to exert selective killing activities on senescent cells [63,64,65]. Interestingly, quercetin has been shown to selectively kill senescent oligodendrocyte progenitor cells and to alleviate amyloid-beta (Aβ) deposition and cognitive decline in animal models of AD [66]. Consequently, many clinical trials are ongoing for senolytic indications of quercetin, especially in combination with the senolytic dasatinib (see http://clinicaltrials.gov (accessed on 10 February 2025); trials number NCT04063124, NCT04685590, NCT04785300, and NCT05422885 for AD and NCT02848131, NCT04733534, NCT05506488, NCT05838560, and NCT06018467 for other indications; last consulted on 10 October 2023). Cell senescence is also very relevant to cancer, and this relation is recently being explored to promote cell senescence in cancer cells and target it by selective senolytics [67,68,69]. However, despite the encouraging results from preclinical and early clinical studies, many translational hurdles still limit the exploitation of these data in clinical practice. Due to the newness of this topic and the very limited natural molecules which are regarded as senolytics (quercetin and fisetin [70,71,72] which are encountered in bee products, are among the very rare and possibly the sole well-studied natural ones), as well as the fact that we are separately detailing nearly all major senescence-related aspects such as DNA damage and repair, epigenetic regulation, inflammaging, autophagy, and cell death in our work, we will not develop this issue here. The combination of many bioactivities against this series of alterations in BP suggests its possible senescence modulatory effects as a propitious avenue. In addition, cumulated evidence until now may pave the way toward a scientific breakthrough in mitigating age-related diseases if the prospection for novel selective senolytics and/or senescence modulators sufficiently succeeds. However, the clinical relevance of senolytics and their “marketed” concept still suffer insufficient solidity, and data about BP compounds in this perspective also suffer limited availability.

We have developed this series of intricate effects of quercetin to give insights on how complex the ferroptosis modulation may be in the case of multitargeting natural compounds, especially those abundantly present in BP and widely known by their highly diversified effects on oxidation, inflammation, autophagy, and cell death. The importance of these mechanisms is pivotal in major age-related diseases such as neurodegeneration and cancer, as is also the understanding and selection of BP-derived candidates in targeting them. The potential of such targeting is undeniably interesting, as it is highlighted by our discussions in the current reviews, but further studies and understanding are warranted before envisioning any real translational and clinical outcomes. We will see other BP compounds more briefly to elucidate their potential in modulating ferroptosis.

In addition to quercetin, other flavanols have been endowed with similar ferroptosis modulatory effects including the contradictory effect in malignant and non-malignant cells. Kaempferol was reported to exert a potent neuroprotective effect even at low concentrations and to potentially prevent experimentally induced oxidative stress-mediated ferroptosis in a mouse hippocampal neuronal cell line [73]. Antioxidant mechanisms were reported to play a pivotal role in the multifaceted ferroptosis preventive effect of kaempferol, as it may be deduced from experimental ferroptosis induction in neurons [39] and other cells such as hepatocytes [74]. Kaempferol also inhibited induced neuronal ferroptosis, and GPX4 upregulation following Nrf2 activation was reported as an underlying mechanism in mouse primary cortical neurons [75]. However, other mechanisms may be equally or more important. As an example, the ferroptosis preventive effect of kaempferol in experimentally injured hepatocytes has also been reported to be mediated by autophagy activation [76]. Myricetin was found to significantly inhibit gastric cancer cells in vitro and in murine xenograft models by promoting ferroptosis in cancer cells [77]. Identified mechanisms in this recent study included mainly the enhancement of NADPH Oxidase 4 (NOX4) by activating its expression and suppressing its ubiquitin-mediated degradation and the subsequent downregulation of Nrf2, which is a major protective mediator against ROS inside malignant cells. Similar results were also recently obtained in mice models of breast cancer, where myricetin effectively suppressed tumor growth through ferroptosis induction [78]. Likewise, this study identified Nrf2/GPX4 pathway downregulation as a main mechanism in the observed effect. In non-malignant cells, myricetin was reported to effectively mitigate ferroptosis in murine models of PD. This recent study found that myricetin-induced effects in dopaminergic neurons (decrease in Fe^2+^, ROS, and MDA, and increase in GSH levels) were reversed by erastatin, a standard ferroptosis activator [79]. All cited studies are very recent, and other BP flavanols remain largely less studied. Isorhamnetin was found to effectively suppress ulcerative colitis by potently inhibiting ferroptosis in intestinal epithelial cells of murine models and in vitro in human and murine cell lines [80]. Rutin was also reported to exert a protective effect against ferroptosis in aging hens, with this protection resulting in ameliorating laying potential, i.e., slowing ovarian aging, of these chickens [81]. It also inhibited ferroptosis in murine models of ventilator-induced lung injury [82]. However, rutin, which is a rutinoside that is quercetin with the hydroxy group at position C-3 substituted with glucose and rhamnose sugar groups linked 1–6, could not be well identified in pollen. The most frequent linkage in pollen is 1–2 between sugars. Despite this structural question, when rutin is taken orally, this linkage is hydrolyzed, and quercetin is liberated for glucuronidation and sulfonation in the liver.

#### 4.1.2. Flavones

Many flavones that are present in BP have also been studied for their modulatory potential on ferroptosis. Apigenin inhibited ferroptosis in the brain of epileptic mouse models [83]. In this study, apigenin was conspicuously revealed by a high-throughput screening technique to be more potent than many known antioxidants, including ascorbic acid and some renowned BP phenolics, in inhibiting intracellular hypochlorite accumulation in vitro. In malignant cells, apigenin was contrarily shown to induce ferroptosis in addition to its known ability to induce cell death through other diverse pathways such as apoptosis, necrosis, autophagic cell death, and senescence [84]. A bioinformatics-based exploration followed by an in vitro study in multiple myeloma cell lines revealed that apigenin exerted a growth-inhibitory activity that was qualified as excellent by authors on these malignant cells [85]. Ferroptosis likely played pivotal roles in the observed effect because cell treatment with ferroptosis inhibitors reduced the apigenin effect by more than three-fold, and a very marked dose-dependent ROS production was recorded inside cells. It is noteworthy that apigenin also induced autophagy, cell cycle arrest, and apoptosis in the studied cell lines, in addition to synergistically potentiating the doxorubicin effect. More significantly, apigenin induced little to no cytotoxicity in normal peripheral blood mononuclear cells isolated from healthy volunteers. Apigenin was also tested in human lung cancer cell lines and showed autophagy and ferroptosis induction with a marked increase in intracellular ROS and MDA levels. These effects were more pronounced when apigenin was included in magnetic nanocarriers [86]. Regarding other diseases, apigenin was reported to inhibit ferroptosis in a rodent hepatocyte cell line with mechanisms including iron uptake suppression and GPX4 activity increase, culminating in potent protection of the studied cell line against chemically induced toxicity [87]. Other mechanisms culminating in ferroptosis inhibition in non-malignant cells and its induction in cancer cells have been reported by a few other studies (reviewed in [88,89]).

Luteolin has also been reported to induce ferroptosis in cancer cells and to mitigate it in non-malignant cells. This molecule triggered ferroptosis in renal cell carcinoma in vitro and in vivo. Involved mechanisms produced classical events that characterize ferroptosis (excessive iron and ROS loads, lipid peroxidation, mitochondrial alterations, and GSH depletion). The authors concluded that luteolin appears to activate heme degradation through excessive upregulation of heme oxygenase 1 (HO-1) to increase iron load [90]. In prostate cancer, luteolin was also found to induce ferroptosis by increasing ferritinophagy through translocating the TFEB into the nucleus [91]. Studies in human cell lines and xenograft models of colon cancer also revealed that luteolin upregulated a tumor suppressor (hypermethylated in cancer 1) gene expression to induce GPX4 suppression and ferroptosis in cancer cells [92]. In the same study, the silencing of this gene restored GPX4 expression and eliminated ferroptosis. It is important to note that luteolin exerted these effects in a synergistic manner with the ferroptosis inducer erastin. Moreover, luteolin could significantly reduce cisplatin-induced ototoxicity by inhibiting ferroptosis in mouse models [93]. In non-malignant cells, many studies reported that luteolin prevented ferroptosis. This was recently evidenced in murine experimental models of myocardial ischemia/reperfusion injury [94], chemically induced liver injury [95], *Staphylococcus aureus*-induced endometritis [96], and chronic atrophic gastritis [97]. In all these models, luteolin manifested many mechanistic traits of ferroptosis inhibition and protected studied organs against experimental injuries. Chrysin, another BP flavone, has also been shown to induce autophagy-dependent ferroptosis and chemosensitization to gemcitabine in pancreatic cancer cells [98] and to inhibit ferroptosis in in vitro and in vivo models of cerebral ischemia/reperfusion injury [99].

#### 4.1.3. Flavanones

BP flavanones have also been endowed with similar ferroptosis-regulating potential. For instance, naringenin inhibited ferroptosis and reduced inflammatory response in cardiomyocytes of murine models of myocardial ischemia/reperfusion injury [100]. This effect, which was abolished by erastin, was explained by the modulation of the Nrf2/System xc-/GPX4 axis. Naringenin was also found to suppress ferroptosis and alleviate immune and inflammatory defects induced by lipopolysaccharides in the chicken thymus, with mitochondrial ROS attenuation reported as the main underlying mechanism [101]. Other conditions where this phytocompound was reported to suppress ferroptosis in animal models included lung epithelial cells in cigarette smoke-induced lung injuries [102] and activated hepatic stellate cells in liver fibrosis [103]. Conversely, in liver cancer cells, naringenin induced ferroptosis in vitro and in vivo and enhanced the efficacy of other ferroptosis-inducing drugs [104]. Naringin, the naringenin-7-*O*-rhamnoglucoside, was also reported to prevent diabetic cardiac autonomic neuropathy in diabetic rats through ferroptosis mitigation [105].

Another flavonoid, this time Hesperetin also exerted similar effects. Using in silico, in vitro, and in vivo assays, a recent study reported that this flavanone effectively enhanced diabetic wound healing mainly by inhibiting ferroptosis and enhancing angiogenesis and endothelial cell viability [106]. In colitis models, hesperetin was also identified as an effective ferroptosis inhibitor in silico, in vitro, and in vivo [107], while it inhibited aflatoxin B1-induced liver toxicity by mitigating ferroptosis in murine models [108]. A recent in silico and in vitro investigation reported that hesperetin promoted ferroptosis in bladder cancer cells and mitigated the latter proliferation and migration [109]. Hesperidin, the hesperetin-7-*O*-rutinoside, was reported to prevent intervertebral disc degeneration by suppressing oxidative stress-induced ferroptosis in human nucleus pulposus cells [110]. Ferroptosis suppression by this phytocompound was also reported as an underlying mechanism in preventing herbicide-induced toxicity in chicken hepatocytes [111]. In malignant cells, hesperidin potently and selectively inhibited ferroptosis induced by the Tribbles pseudokinase 3 oncogene, an effect that resulted in efficient inhibition of tumorigenesis in diverse head and neck squamous cell carcinoma human cell lines [112].

Pinocembrin, another flavanone reported in BP, was found to protect animal models against intervertebral disc degeneration by suppressing ferroptosis in the cartilaginous endplate chondrocytes. The main unveiled mechanism was the upregulation of Nrf2 [113].

#### 4.1.4. Other Flavonoids

Anthocyanidins are also frequent BP compounds, with the glycosylated forms (anthocyanins) of cyanidin and delphinidin being among the most reported [5]. Through ferroptosis suppression, cyanidin-3-*O*-glucoside, one of the most common forms, mitigated ischemia-reperfusion injury in a myocardial murine cell line [114] in kidney tubular cells in vitro (human cell line) and in vivo (mice) [115]. Cyanidin chloride was also found to be a potent ferroptosis inhibitor, through strongly inhibiting lipid peroxidation and mitochondrial damage, when investigated for doxorubicin-induced cardiotoxicity in zebrafish [116]. The differences in effect mechanisms and extents between anthocyanidins and their conjugated forms remain, however, not studied.

Catechins, including epicatechin, have also been reported to inhibit ferroptosis in the brain by reducing ferrous ion load and lipid peroxidation levels [89].

### 4.2. Phenolic Acids

Derivatives of both benzoic and cinnamic acids are known to be ubiquitous in BP. From these two subfamilies, numerous molecules have been identified as substantial ferroptosis modulators.

#### 4.2.1. Benzoic Acids

Many BP benzoic acids were studied for their effects on ferroptosis. Gallic acid was especially studied in ferroptosis induction in cancer apparently due to the substantial evidence that has been cumulated on its anticancer potential. We found one study about non-malignant neurological conditions. In murine models of neuropsychiatric disorders culminating in chronic pain and depression, gallic acid was able to markedly prevent ferroptosis in the spinal cord and, in addition to increasing GPX4 and glutathione levels and reducing MDA and ROS levels, alleviated diverse pathological marks related to the experimental disease [117]. It is noteworthy that many studies, although not specifically assessing ferroptosis, have reported that gallic acid exerted many ferroptosis-preventing effects in non-malignant conditions inside the CNS. In rat mania models, it increased total glutathione peroxidase and other antioxidant enzymes and decreased ROS, nitrite, and thiobarbituric acid reactive substances (lipid peroxidation byproducts) levels in the cerebral cortex, hippocampus, and striatum, in addition to reducing other neurological alterations such as hyperlocomotion and cholinergic transmission defect [118]. In other models of experimentally induced neurotoxicity, gallic acid increased intracellular levels of glutathione peroxidase and other antioxidant enzymes and reduced lipid peroxidation in brain tissues [119,120]. Moreover, a very recent study reported that a synthetic hybrid molecule combining gallic acid and a cyclic dipeptide produced an effective ferroptosis inhibition and a suppression of the toxic interplay between ferroptosis and aberrant aggregation-prone proteins involved in AD, namely Aβ and tau [121].

Regarding ferroptosis induction, a recent study showed a ferroptosis-mediated anticancer effect of gallic acid in human breast cancer and melanoma cell lines. This study reported an intriguing observation. While a low-level laser was unable to modify cell survival in any tested cell line, cell pre-treatment with this laser and then their treatment with gallic acid was more efficient in killing cancer cells than the cell treatment with gallic acid, followed by their laser treatment. Moreover, gallic acid alone induced a marked decrease of cell viability in both cancer cell lines but only induced a slight decrease at high doses in normal (non-tumorigenic breast epithelial and healthy dermal fibroblast) cell lines [37]. Increasing lipid peroxidation and total ROS production and decreasing GPX4 activity in tested cancer cell lines were attributed to gallic acid. Interestingly, this BP ubiquitous phenolic acid was found to exert a marked promoting effect on ferroptosis, necroptosis, and mitochondrial-mediated apoptosis at the same time in human cervical and neuroblastoma cell lines [122]. More importantly, this effect of gallic acid was not abolished by downstream inhibitors of the three cell death pathways. The authors suggested that additional mechanisms may drive the cell death-promoting effect of gallic acid. To further raise attention, it has been recently reported that gallic acid induced these three cell death pathways in a timely manner. Involving diverse metabolic pathways, this phytocompound induced ferroptosis at the early stage, apoptosis at the middle stage, and necroptosis at the late stage of the cell death process in a cervical cancer cell line [123]. A very recent study confirmed the potential of gallic acid against hepatocellular carcinoma in vitro. This acid markedly reduced cell viability and migration by inducing ferroptosis in the studied cell line [124]. The revealed mechanism of this effect was the inhibition of the Wnt/β-catenin pathway. Another recent study found that gallic acid inhibited the expression of all the identified prognosis marker genes related to ferroptosis in human colorectal cancer. Among the identified colorectal cancer hub genes that were regulated by gallic acids, the sigma-1 receptor (involved in antioxidant defense and its depletion result in depleted GPX4), tumor protein p53, and GPX4 proteins had, in descending order, the highest gallic acid binding potential according to molecular docking [125]. The anti-ferroptosis potential of this phenolic acid has been verified by molecular docking and further confirmed in a human colorectal carcinoma cell line in the same study [125].

Another phenolic acid found in pollens, ellagic acid, induced ferroptosis in activated hepatic stellate cells of murine models (death of these cells is considered an efficient strategy to prevent hepatic fibrosis) [126]. This effect was observed along with increased levels of iron accumulation and lipid peroxidation and depletion of glutathione and GPX4 inside these cells, as well as a decrease in plasma membrane abundance of ferroportin, an iron transporter that translocates iron from cytoplasm to extracellular space. Ferroptosis induction was further confirmed by the authors in a human hepatic stellate cell line. Another study reported that ellagic acid reduced thiobarbituric acid reactive substances and increased antioxidant enzymes and glutathione levels in the brains of murine models of AD [127].

Other benzoic acids that are repetitively reported in BP were studied to a lesser extent. Vanillic acid mitigated ulcerative colitis in vitro (human cell lines) and in vivo (mice) by significantly suppressing ferroptosis in intestinal epithelial cells [128]. Protocatechuic acid was also endowed with similar protective effects, namely ulcerative mitigation by inhibiting ferroptosis, in a recent study with similar models [129]. Syringic acid alleviated lower limb ischemia/reperfusion injury in mouse models by suppressing ferroptosis in skeletal muscle cells [130]. Although classical biomarkers of ferroptosis and inflammatory response were significantly reduced in the three studies, some different mechanisms that we could not develop here were, however, reported for these distinct phenolic acids.

#### 4.2.2. Hydroxycinnamic Acids

Some hydroxycinnamic acids that are present in diverse BPs have also been studied for their ferroptosis-modulating potential. Hybrids made between flavonoids, including quercetin and cinnamic acid, through amide bonds (which are more stable than ester bonds) were found to be effective against many neurodegeneration traits in in vitro (mouse hippocampal cell line) and in vivo (AD mouse) models. The authors reported that ferroptosis countering was among the involved mechanisms [131]. Another study reported that similar hybrids of quercetin with cinnamic or ferulic acid expressed potent ferroptosis inhibition [132]. A *p*-coumaric acid nano-formulation was also reported to potently inhibit chondrocyte ferroptosis in murine models of temporomandibular joint osteoarthritis [133]. Caffeic acid was reported to reduce muscular atrophy in chronic kidney disease murine models [134]. The authors identified ferroptosis inhibition as an underlying mechanism but did not exclude other mechanisms in the observed effect. Caffeic acid was also found to alleviate cerebral ischemia/reperfusion injury in rats by suppressing ferroptosis via Nrf2 activation [135]. Chlorogenic acid, the caffeic and quinic acids ester, was also reported to protect against hypoxic-ischemic brain injury by inhibiting ferroptosis in hippocampal and cortical neurons of a newborn mouse model [136]. Ferroptosis inhibition was also unveiled as a mechanism for alleviating chronic stress-induced duodenal injury in mice models [137]. Rosmarinic acid, delivered in nanoliposomes, protected mice models against ischemic stroke by suppressing ferroptosis in brain tissues [138]. This hydroxycinnamic acid reduced brain iron deposition induced by ischemia reduced ROS load, and normalized GPX4 levels and mitochondrial structures in BBB microvascular endothelial cells. Another study reported that rosmarinic acid mitigated bronchial epithelial cell ferroptosis induced by septic-associated lung injury in vitro and in vivo [139]. Ferulic acid was reported by many studies to mitigate ferroptosis in diverse experimental conditions, including gamma-radiation-induced liver injury [140], myocardial ischemia-reperfusion injury [141], and iron-induced pancreatic β cell death [142]. In the nervous system, ferulic acid was not studied in age-related neural alterations such as NDD but was reported to prevent cognitive dysfunction through ferroptosis mitigation in Wilson’s disease (a treatable genetic disease characterized by copper overload and manifesting neurological, psychiatric, and other disorders) [143]. One study evaluated ferulic acid in esophageal squamous cell carcinoma and reported that it mitigated the growth and invasion of malignant cells by inducing their ferroptotic death [144]. Observed mechanisms resulted in all the major ferroptosis traits, including MDA, ROS, iron loads, and depletion in antioxidant defense (GSH, superoxide dismutase, and GPX4 activities).

### 4.3. Stilbenoids

Resveratrol was reported to inhibit ferroptosis by upregulating GPX4 and glutathione and reducing intracellular levels of ROS and Fe^2+^ in animal models of neurodegeneration [145]. Its inhibition of ferroptosis was also confirmed in vitro in primary cortical neurons isolated from neonate rats and in vivo in adult rats either submitted to oxygen-glucose deprivation/reoxygenation- or chemically induced ferroptosis [146]. Pretreatment with resveratrol significantly reduced intraneuronal iron and ROS, mitochondrial alteration, degenerating neurons, brain ischemic damage, and infarction volume and enhanced ferritin, GPX4, and glutathione levels. In neonate murine models of ischemic brain injury, resveratrol suppressed ferroptosis and upregulated the SIRT1/Nrf2/GPX4 pathway, which correlated with a decrease in iron load, lipid peroxidation, and mitochondrial alterations in hippocampal neurons, a correction of brain atrophy and other damages, and restoration of learning and memory faculties [147]. The upregulation of Nrf2/GPX4 and SIRT1/GPX4 were also reported in animal models of spinal cord [148] and brain [149] injuries, respectively. This evidently correlated with the prevention of ferroptosis-mediated neuronal death in both studies and other intracellular hallmarks, i.e., reduction of intracellular iron load, lipid peroxidation, and mitochondrial alterations. In a mouse hippocampal neuronal cell line, resveratrol and quercetin separately inhibited experimentally induced ferroptosis both in wild-type and in Nrf2-knocked-down cells and produced a significant decrease in intracellular ferrous ion accumulation and lipid peroxidation without increasing intracellular glutathione levels [150]. This recent study supports the existence of diverse mechanisms in resveratrol-, quercetin-, and possibly other polyphenol-mediated anti-ferroptosis effects that may not always rely on their confirmed antioxidant potential. Further studies to verify and understand such possibilities will hence be of great importance in targeting ferroptosis.

In cancers, many recent studies reported that resveratrol promotes ferroptosis in malignant cells. Genetic profiling-based prediction and molecular docking revealed resveratrol as one of the best candidates from 27 herbal medicines to bind selected ferroptosis-related genes in gastric cancer [151]. To confirm these results, this study verified the presumed effects in vitro. ROS-dependent induction of ferroptosis by resveratrol was reported in acute myeloid leukemia [152] and colorectal cancer [153] cell lines. Ferroptosis was reported as the possible death mechanism induced by resveratrol in other malignant cell lines [154]. A study of different head and neck cancer cell lines reported that resveratrol, through inducing SIRT1, modulated the epigenetic programming in the epithelial-to-mesenchymal transition process (EMT, an important cell transformation especially toward cancer cell invasion), thereby promoting malignant cell transformation toward mesenchymal phenotype which is more sensitive to ferroptosis [155]. This effect mediated a proper significant reduction of cancer cell survival as well as a potentiation of another ferroptosis inducer by resveratrol. In addition to the direct induction of ferroptosis in cancerous cells, this study, therefore, suggests another novel interventional method of anticancer therapies through epigenetic modulation of ferroptosis. We will discuss the very important epigenetic modulatory potential of phenolics and other BP compounds in an upcoming subsection. A ferroptosis-mediated potentiating effect by resveratrol in cancer treatment was also verified in cancer immunotherapy by enhancing the cytotoxic effect of CD8^+^ T lymphocytes in lung squamous cell carcinoma [156]. This study combined proteomic, bioinformatic, in vitro, and in vivo investigations and revealed a potent reduction of malignant cell viability through classically known ferroptosis mechanisms.

### 4.4. Phenolamides

Phenolamides (or phenylamides), the very ubiquitous conjugates of phenolic acids and mono-polyamines, have been revealed by recent studies to be among major BP compounds and to surpass polyphenol content in many BPs. We have recently reviewed their examples and structures [5]. Studies in the plant kingdom revealed that these secondary metabolites participate in plant resistance to diseases and adaptation to environing, biotic and abiotic stress, as well as in other vital functions such as reproduction [157]. Although phenolamide bioactivities remain scarcely studied, some polyamines that are present in all living cells, e.g., spermidine, are widely studied and marketed. Phenolamides are generally endowed with potent antioxidant and anti-inflammatory bioactivities [158,159], and some phenolamide dimers have been particularly reported to possess strong iron chelating potential [159]. However, studies on their ferroptosis modulatory effects remain absent.

Polyamines, in contrast, have been investigated in ferroptosis by few studies. Although polyamine levels generally decrease with age, spermidine levels have been shown to decline with age in mice but do not correlate in terms of age with humans, suggesting that other factors, such as diet and microbiota, may be more influential in regulating these levels [160,161]. More intriguingly, some studies reported that spermidine and spermine levels in 90 years old humans are higher than in 31–80 aged individuals [161]. Considering these observations, targeting ferroptosis mechanisms with polyamines and possibly with phenolamides should be carefully appraised, and more studies are needed to illustrate suitable interventions in malignant and non-malignant pathologies. Personalized considerations of pathophysiological milieus may be crucial in this context. Anyhow, increased polyamine levels are firmly regarded as a promoting factor of tumor growth, invasion, and metastasis [162,163,164]. Intracellular levels of polyamine are determined by their biosynthesis, transport, and catabolism, in addition to dietary intake and other factors such as gut microbiota-mediated synthesis [161,162]. Keeping balanced amounts of polyamines inside a cell is crucial for its survival. An excessive content of polyamines may drive rapid anarchic cell growth and oncogenesis, while their depletion leads to a complete blockade of cell growth and proliferation [161].

Spermidine/spermine N1-acetyltransferase 1 (SSAT) is the rate-limiting and responsible enzyme for polyamine catabolism but is also a contributor to generating glutathione pool by enhancing glutamine conversion to glutamate [165]. SSAT, which is encoded by the *SAT1* gene, has been recently linked to ferroptosis regulation in cancer cells, although exact mechanisms remain unknown [166]. SSAT plays a crucial role in responding to oxidative and inflammatory stimuli [166]. Moreover, the p53 protein can increase SSAT through transcriptional activation of *SAT1* to result in excessive lipid peroxidation and ferroptotic cell death [167]. Polyamine-mediated targeting has recently been pursued as a valuable strategy to induce ferroptosis in cancer cells [168]. SSAT suppression has been reported to inhibit ferroptosis in cancer cells [166]. Pharmacologically induced GSH depletion in combination with SSAT-mediated activation of polyamine catabolism showed efficient suppression of tumor growth in vitro and in xenograft models [165]. Spermidine has been reported to promote ferroptosis in cancer cells and to sensitize these cells to chemo- and immuno-therapies [169,170]. Studies are still scarce, with difficult-to-decipher results, and some reports indicated that spermidine appears to tackle the neo-oncogenic process but to promote the proliferation of already-formed tumors [169]. In turn, spermidine decreased p53 levels and downregulated SAT1 expression, resulting in ameliorating AD pathology in murine models through mitigating ferroptosis [171]. Spermidine also tackled ferroptosis through the SLC7A11/GPX4 pathway in in vitro models of non-alcoholic fatty liver disease [172], in addition to preventing diverse cardiomyopathic alterations in diabetic mice models via mechanisms including ferroptosis suppression [173]. It is important to keep in mind that spermidine while being reported to be altered in non-malignant pathological conditions like these examples, is known to be a very pleiotropic bioactive, and deciphering the extent of ferroptosis modulation in its bioactivities needs further investigation.

### 4.5. Carotenoids

Carotenoids and retinol metabolites have also been linked to ferroptosis inhibition. Lycopene inhibited chemically induced ferroptosis in the mice hippocampus through upregulating GPX4 and other ferroptosis-limiting proteins and reducing lipid peroxidation, ROS accumulation, glutathione depletion, neuronal iron load, and mitochondrial alterations [174]. In addition, it ameliorated cognitive and other neuronal alterations and dramatically reduced neuronal loss. Lutein is metabolized to 3′-epilutein and 3′-oxolutein in the human body. These metabolites inhibited glutamate-induced ferroptosis in a neuronal cell line by reducing intracellular iron and ROS loads, increasing antioxidant enzymes, and downregulating the expression of diverse lipid peroxidation-promoting genes [175]. β-carotene and vitamin A have been reported to inhibit ferroptosis through their antioxidant potential, especially lipid peroxidation inhibition, and are, importantly, both endowed with a high potential to cross the BBB [89,176]. Vitamin A and its metabolites have been shown to inhibit experimentally induced ferroptosis in neuronal and non-neuronal cell lines [177]. This effect was more potent than α-tocopherol, which is regarded as the canonical anti-ferroptosis vitamin, and was interestingly induced in cancerous cell lines [177]. Hence, retinoids, including retinol, retinal, and retinoic acid, are known to encompass ferroptosis-inhibitory potential, but they tend to lose it when they are metabolized by retinol saturase (an oxidoreductase, which may, therefore, act as ferroptosis promoter [178]. This metabolism has been recently proposed as a therapeutic target to induce ferroptosis in cancer cells [178].

### 4.6. Vitamins

Many nutrients, including vitamins and minerals that are widespread in BP from different geographical and botanical sources, have also been identified as important ferroptosis modulators. Dual ferroptosis modulation (inhibition and induction) depending on the cellular context has also been verified for some of them, as we will see hereinafter.

#### 4.6.1. Vitamin C

Vitamin C is intricately involved in ferroptosis regulation. When vitamin E detoxifies fatty acids, it is oxidized to an α-tocopheroxyl radical and is reduced back to its antioxidant form by vitamin C (in the absence of vitamin C, vitamin E has been reported to promote lipid peroxidation instead of inhibiting it) [179]. On the other side, vitamin C has been reported to induce ferroptosis in cancer cells [180] and to sensitize these kinds of cells to ferroptosis inducers [181]. Vitamin C was found to significantly inhibit thyroid cancer cells through ferroptosis that manifested in triggering ferritinophagy, subsequently resulting in increased intracellular load of irons and ROS and GPX4 inactivation [180]. Vitamin C also induced melanoma cell death in vitro and in vivo through ferroptosis triggering [182]. Ferroptosis induction was also involved in overcoming the resistance of non-small cell lung cancer to Osimertinib and significantly suppressing the metastasis in human patient-derived xenograft murine models [183]. The synergism between vitamin C and Osimertinib strikingly resulted in a 91.39% tumor inhibition rate, but it is worth noting that the association was delivered in nanocarriers encompassing a nano-catalytic sensitizer to maintain ferrous catalytic activity to induce Fenton reaction inside the malignant cells. Such a synergism exerted by vitamin C was even reported in highly resistant tumors such as glioblastomas where the oxidized form of this vitamin, the dehydroascorbic acid, resulted in a strong ferroptotic cell death when combined with another ferroptosis inducer, viz. erastin, in mouse models of brain tumors [184]. The cell death rate induced by the association was dramatically greater than that induced by the ferroptosis inducer alone. More appealingly, the ferroptosis-mediated killing of malignant cells by pharmacological doses of vitamin C was recently found to not be suppressed by ferroptosis inducers, suggesting that vitamin C may interestingly have other direct triggering mechanisms of ferroptosis in cancer cells [184]. Vitamin C is known to mediate the reduction of ferric ions (Fe^3+^) to ferrous ions (Fe^2+^) and increase the availability of ferrous pool, and, especially at high doses and in glycolytic environments such as cancers, to promote glutathione depletion through its oxidative dimerization [181,185,186]. The altered metabolism in malignant cells can, thus, presumably be targeted by vitamin C [186]. In murine normal cells, namely pancreatic β-cells and embryonic fibroblasts, vitamin C was shown to prevent chemically induced ferroptosis concomitantly with increasing glutathione levels and reducing lipid peroxidation and ROS accumulation [181]. Selective killing of cancerous cells by vitamin C, such as in the case of pancreatic cancer, where ferroptosis induction was an underlying mechanism, has been reported in some studies, but this remains poorly understood [181]. The duality of ferroptosis modulation by vitamin C is, therefore, intricate and depends on the cell type and many biochemical interactions that we cannot develop here. Another noteworthy remark is that blue light has been shown to markedly promote ferroptosis induction in cancer cells and to increase intracellular levels of vitamin C, even at low doses. A recent study that reported these results revealed that blue light resulted in a 20-fold reduction of the administered dosage of vitamin C while maintaining a high cellular ferrous load and an effective melanoma elimination and prolonged survival in animal models [182]. The intriguing results reported by novel formulations and combinations of therapeutic means to target ferroptosis warrant attention and must be tested with other BP compounds that we have seen to encompass interesting ferroptosis-modulating potential.

#### 4.6.2. Vitamin E

Vitamin E inhibited experimental ferroptosis by suppressing polyunsaturated fatty acid oxidation through lipoxygenase inhibition in cortical neurons of murine epileptic models [187]. In addition, vitamin E detoxifies lipid radicals and, therefore, stops the formation of phospholipid hydroperoxides [188]. These action mechanisms are of great importance since lipid peroxidation can either occur enzymatically (e.g., by lipoxygenases) or non-enzymatically through direct free radical-mediated induction [189]. Note that polyunsaturated fatty acids are the targets of the cell-killing effect of lipid peroxidation [7], which is a pathological marker of almost all NDDs [145]. Due to its notable potential in inhibiting lipid peroxidation, vitamin E is a well-recognized endogenous inhibitor of ferroptosis [176,190], and this has been shown to mediate advantageous effects in some clinical and preclinical studies of NDD [191,192]. Vitamin E’s preventive effect on seizure induction and severity in animal models of epilepsy was shown to be mediated by its ferroptosis inhibitory effect, as some studies verified in cortical [193] and hippocampal [187] neurons by administering known ferroptosis inducers. Evidently, more mechanisms may be involved in this preventive effect and need to be verified by other studies. Nevertheless, vitamin E administration to epileptic animals resulted in decreased levels of lipoxygenase 15, MDA, and iron and increased levels of GPX4 and glutathione in hippocampal tissues [187]. Vitamin E has been reported to suppress ferroptosis but no other forms of cell death [194], which may endow it with greater importance in some cases, such as selective targeting. More specifically, a study in motor neurons derived from human induced pluripotent stem cells found that their treatment with a GPX4 inhibitor resulted in the death of almost all neurons and that this death was prevented by only one antioxidant among those tested, which was the vitamin E acetate [192]. Vitamin E was also found to prevent cytotoxicity and induced ferroptosis in hypoxic ischemia of ferret neonates, an effect that was shown to be driven by antioxidant potential, but also with other mechanisms as it was more pronounced than that of a ferroptosis selective inhibitor [195]. Vitamin E has been frequently found to suppress ferroptosis through correcting diverse ferroptosis hallmarks in the CNS (other examples than those that we have cited exist), but results about neurodegeneration-suppressing outcomes from clinical trials were controversial, although vitamin E has been frequently used to successfully mitigate neurodegeneration events in animal models and clinical studies [194]. The inconsistency of clinical trial results is a universal problem in novel therapeutic approaches, especially in natural products. Numerous shortfalls may stand behind these discrepancies, including incorrect or insufficient sampling, non-integrative pathophysiological considerations, unfitted standards in considering patient and disease variabilities, inadequate relevancy of assessed parameters, etc. Further critical approaches and large-scale studies are, therefore, needed. Pathophysiological mechanisms linking ferroptosis to neurodegeneration are still poorly understood, as are possible intervention mechanisms of vitamin E and other antioxidants in ferroptosis.

In contrast to what we have seen for vitamin C and many phenolic antioxidants, a dual inductor-inhibitory effect of vitamin E on ferroptosis has not been verified, at least to the current level of knowledge. Indeed, vitamin E has been found to exert a pro-tumorigenic effect by suppressing oxidative ferroptosis marks triggered by the iron carrier transferrin knockdown in circulating tumor cells originating from patients with metastatic melanoma [196]. Transferrin mediates iron pool, ROS, and lipid peroxidation lowering inside circulating tumor cells and, thus, induces the resistance of these cells to ferroptosis and strengthens their metastasis-inducing potential [196]. Despite the widely reported anticancer potential of vitamin E by preclinical [197,198] and clinical [199,200,201] studies, such action mechanisms must be considered depending on the disease’s pathophysiological context. These mechanisms, among others (see good examples in [202] for metabolomic analysis results and in [203] for genetic analysis), may stand behind some reports (e.g., [204]), implicating the positive correlation between vitamin E circulating levels and the risk of some cancers.

### 4.7. Minerals

In addition to their experimentally verified effect on ferroptosis, some minerals have been identified as core ferroptosis actors by participating in the structure of key ferroptosis mediators and effectors, in addition to their known roles in the aging process and in age-related diseases.

#### 4.7.1. Selenium

Selenium, abundantly present in some BPs [4], is a pivotal element in ferroptosis regulation. It is a crucial component of selenoproteins (25 are known in humans, including GPX4) by being present in selenocysteine, which is a rare amino acid that determinates the effect of GPX4 and is present in its active site (blocking the incorporation of selenocysteine in translational genesis of selenoproteins induces mice embryonic death at the same levels of that noted in *Gpx4* knockout ones) [205]. Selenium supplementation resulted in enhanced GPX4 expression and increased resistance to ferroptosis in cell cultures and animal models [205]. GPX4 upregulation was reported as a likely underlying mechanism by which sodium selenite suppressed neuronal and oligodendrocyte ferroptosis and recovered locomotor activity in murine models of spinal cord injury [206]. Upregulation of the Nrf2/GPX4 pathway was also found to drive the ferroptosis-inhibiting effect of selenium in autistic animal models, which was accompanied by suppressing diverse neuronal damages and ameliorating behavioral and cognitive defects [207]. Selenium supplementation was also found to alleviate drug-resistant epileptic seizures in children suffering a decline in GPX activity [208]. Multiple studies in animal models have reported that selenium protects against ferroptosis in NDD, such as AD and PD, which are characterized by brain selenium depletion [209]. Selenium supplementation decelerated cognitive deterioration of AD patients in some clinical trials [194]. Chronic accumulation of oxidation- and iron accumulation-related defects are not the sole case where selenium may have a preventive role of ferroptosis in the neurodegeneration process. Presenilins, which are depleted due to mutations in familial forms of AD, have been shown to suppress ferroptosis promotion and more extensively suppress GPX4 expression by regulating selenium supply to the cells via selenoprotein P, which delivers it to the low-density lipoprotein receptor-related protein 8 (LRP8) which, when knocked out in mice, reduces brain selenium by more than 50% and results in severe neurodegeneration [210]. Accordingly, selenium administration to mice presenting a triple mutation in presenilin-1, amyloid precursor protein (APP), and tau resulted in suppressing memory, learning, and behavioral impairments [210]. Moreover, a very intriguing possibility has recently emerged from preclinical evidence implying that Aβ and tau pathological depositions may be epiphenomena of ferroptosis induction [211,212], a relation where selenium may play a crucial role as reported by many experiments [211].

Sodium selenite was recently found to selectively induce ferroptosis in diverse human cancer cell lines (e.g., breast, colon, glioma, neck, prostate) by impairing cystine import and glutathione homeostasis and inducing intracellular superoxide and iron accumulation [213]. This selenium salt, at high doses, induced ferroptosis in a human cell line and an animal model of ovarian cancer by selective inhibition of GPX1 and GPX4 expression, along with presenting no toxicity towards normal cells in other organs (liver and lungs) and not inducing toxicity-mediated animal death [214]. Selenium compounds have been reported to exert both oxidant and antioxidant bioactivities depending on their concentrations and chemical formulae [214]. In addition, several ferroptosis-related vulnerabilities involving selenium metabolism, particularly selenoproteins, have been recently identified in cancers. Selenophosphate synthetase 2, an enzyme involved in selenocysteine biosynthesis, has been found to be selectively essential for the survival of malignant cells but not in normal cells, especially in cancers that rely on GPX4 to protect cancer cells against ferroptosis such as breast cancer [215]. The cystine-glutamate antiporter xCT, which is involved in selenium uptake and cystine importation for glutathione synthesis and, thus, prevents ferroptosis by promoting GPX4 expression and activity, was found to be particularly crucial for breast cancer cells, contrarily to normal breast cells [216]. In another study, LRP8 was identified as a resistance factor in cancer cells, and its depletion was accordingly found to strongly reduce GPX translation by causing early ribosomal stalling and subsequent proteasome-mediated proteolysis of nascent GPX4 [217]. These examples, and others that were not explained here [218,219,220], open new ways to novel selective therapeutic targeting of selenium metabolism in specific cancer cells and to novel forms of selenium use to target carcinogenesis processes and explore its vulnerability to oxidative regulation.

Selenium at high doses may present safety issues or compromise anticancer therapies. Selenium has a narrow interval between therapeutic and toxic dosages [213]. Excessive exposure to this microelement was reported as a risk factor for PD [221]. In another study, selenium has been found to protect cancer cells from ferroptosis mechanisms induced in vitro by pharmacological doses of vitamin C by antioxidant selenoenzymes (interestingly, not including GPX4), while in vivo investigation showed that selenium deficiency enhanced cancer cell sensitivity to high doses of ascorbate as evidenced by human-derived glioblastoma xenografts [222]. Despite the abundant and encouraging evidence for the possible use of selenium administration and/or physiological modulation in tackling neurodegeneration and carcinogenesis processes, further studies are needed, specifically to define dosage and forms of use as well as determinant factors that may also depend on disease context.

#### 4.7.2. Zinc

Zinc has been unveiled to be crucial for ferroptosis by recent studies which observed that a zinc chelator suppressed ferroptosis and zinc addition promoted ferroptosis, even when an iron chelator was co-administered [223]. Zinc is a very pleotropic mineral and appears to hold paradoxical roles toward redox processes as well as toward ferroptosis [224]. The zinc transporter solute carrier family 39 member 7 (Zinc Transporter SLC39A7, or ZIP7), which controls zinc transport from the endoplasmic reticulum to the cytosol, was identified as an essential factor in ferroptosis. ZIP7 chemical inhibition or *SLC39A7* knockdown conferred strong anti-ferroptosis protection, which was abolished by zinc supplementation in human cancer cell lines [223]. Zinc is a key component of more than 3000 zinc-finger transcription factors and a critical cofactor for over 300 enzymes, while diverse studies reported that, depending on the dosage and administration form, zinc may either decrease or increase glutathione, lipid peroxidation, and ROS accumulation [225]. Treatment of different cancer cell lines, including breast, kidney, and non-small cell lung cancer cells, with high levels of zinc-induced ferroptosis, were marked by an increase in ROS accumulation and lipid peroxidation, a glutathione and GPX4 depletion, and an iron homeostasis alteration [226]. Ferritinophagy appears to also play an important role in zinc-mediated ferroptosis. This NCOA4-dependent autophagy was found, at least partly, to mediate ferroptosis that was observed in human vascular endothelial cell lines and animal models after exposure to zinc oxide nanoparticles and was reversed by a selective ferroptosis inhibitor [227]. Exposition to zinc oxide nanoparticles was also found to induce ferroptotic neuronal death in animal models [228].

Zinc is also known to be deeply involved in CNS functions and neurodegenerative processes. The concentration of this vital trace element in the brain is higher than that in other body organs [212]. Although its roles are still largely debated, zinc is present at high levels in AD and PD patient brains and was shown to alter BBB integrity, drive mitochondrial and neuronal damage and death, be tightly linked to aberrant protein deposition and neuroinflammation, and possibly be involved in the neurodegenerative process as early as its early stages [212,229]. Zinc is highly involved in synaptic transmission, plasticity, and long-term potentiation, but its astounding flux in synapses declines with age, suggesting a role in cognitive decline and other neurodegenerative events [211]. Zinc was found to suppress ferroptosis in animal models of spinal cord injury [230]. This study reported that zinc gluconate injection to animals resulted in a strong upregulation of the Nrf2/heme oxygenase-1 axis and in increasing GPX4 and glutathione levels and reducing ROS and lipid peroxidation byproduct levels. Concomitantly, a marked reduction of mitochondrial injuries, inflammatory markers, and overall spinal cord injury was observed. Ferroptosis modulation appears, therefore, to be endowed with a novel therapeutic targeting avenue by modulating zinc availability and pharmacokinetics, either through inhibiting zinc-mediated ferroptosis in neurodegeneration pathophysiological processes or selectively inducing it in cancerous cells. This research avenue is still very recent; further studies are needed even at the experimental level.

### 4.8. Other Promising Compounds

Diverse other BP compounds have been verified to exert important ferroptosis modulatory effects with supposed implications in neurodegeneration, cancer, and other age-related diseases. Uronic acid, a polysaccharide reported to be abundant at high concentrations in a *Lycium barbarum* BP extract [231], was found to act as a ferroptosis promoter in ovarian cancer cells by modulating p53 signaling [232]. Diverse arguments may support the presence of ferroptosis inhibitory effects of BP polysaccharides. First, natural polysaccharides from diverse botanical sources were reported to strongly inhibit ferroptosis by diverse mechanisms [233]. Second, inflammatory and oxidative processes are tightly linked to ferroptosis incidence, as we have already seen. Third, polysaccharides from some BPs have been shown to have important antioxidant and anti-inflammatory activities in addition to other bioactivities diverse [35]. Consequently, possible ferroptosis inhibition of these abundant BP compounds may be an interesting research avenue.

Other nutrients that are known as universal BP compounds are involved in ferroptosis regulation but could not be discussed here due to their very expanded and complex nutritional roles and biochemical interactions. This includes, for example, unsaturated fatty acids and other lipidic compounds that participate in cell membrane structures and functions, diverse amino acids such as cysteine, glucose and its sources, and minerals such as iron. Good reviews on these nutrients can be found in [22,205,234]. Membranes with high contents of polyunsaturated fatty acids appear to be more prone to ferroptosis, especially when those with two bisallylic groups (e.g., arachidonic acid) are more abundant than those with one bisallylic group (e.g., linoleic acid) [7]. The last type of fatty acids is the most abundant in BP, according to many studies [235,236]. Monounsaturated fatty acids were also found to limit membrane lipid peroxidation, but the underlying mechanisms remain unclear [7].

Coenzyme Q10 (ubiquinone, a name which evokes its ubiquitous presence in cell membranes) is another physiological molecule that, beyond its classical known vital roles in adenosine triphosphate synthesis and other mitochondrial functions as well as in antioxidant defense, plays critical roles in preventing ferroptosis, a role which appears to mainly emanate from regulating lipid peroxidation in plasma membrane [22,205,237]. Mitochondria ensure coenzyme Q10 synthesis and its transport to the plasma membrane [237], and both mitochondrial fitness and coenzyme Q10 intracellular levels are known to decline with age and in aging-related diseases [237,238]. This decline may likely be another factor in driving age-related proneness to cell death triggers, including ferroptosis. Ubiquinone is present in some BPs at substantial amounts that surpass its levels in a great number of its most rich natural sources [239]. Ferroptosis suppressor protein 1 (FSP1) is a crucial factor in resisting ferroptosis by reducing coenzyme Q10 to trap free radicals and mediate a GPX4-independent and potent pathway to inhibit ferroptosis [240,241]. Pharmacological inhibition of this axis has been found to induce radiotherapy sensitivity in kelch-like ECH-associated protein 1 (KEAP1)-deficient lung cancer (a frequent subtype of lung cancer that resists chemotherapy and radiotherapy) through inducing ferroptosis [242]. The revealed underlying mechanism was that KEAP1 drives Nrf2 degradation in proteasomes and markedly upregulates FSP1. KEAP1 deficit in lung cancer cells, therefore, resulted in enhanced antioxidant defense and resistance to ferroptosis inside these cells. The coenzyme Q10/FSP1 axis was also found to mediate resistance to ferroptosis in human hepatocellular carcinoma and was successfully targeted (inhibited) in human tumor samples to induce potent ferroptosis, promote anticancer immune response, and effectively suppress tumor growth [243]. Similarly, diverse coenzyme Q10 inhibitors have been shown to trigger ferroptosis, and dietary supplementation of this coenzyme has been shown to prevent lipid peroxidation [22,244]. Coenzyme Q10 administration to hypobaric hypoxia animal models prevents ferroptosis, which was triggered by hypoxia-resulting formaldehyde accumulation [245]. Inhibition of coenzyme Q10 reduction was also reported to participate in preventing ferroptosis in murine neuronal cells [246]. However, despite these results, coenzyme Q10 supplementation failed to reverse chemically induced ferroptosis in some *FSP1*-silenced cells, suggesting that other mechanisms must be sought in FSP1-mediated protection against ferroptosis [247]. Selective inhibition of GPX4-mediated glutathione synthesis and FSP-mediated coenzyme Q10 reduction using nano-catalytic particles was successfully used to induce pronounced ferroptotic cell death in chemotherapy-resistant breast cancer cells and animal models [248]. Anyway, targeting the FSP1/Coenzyme Q10 axis to induce ferroptosis in cancers, especially those that do not rely on GPX4-related mechanisms to tackle ferroptosis, appears to be a promising strategy [247,249,250]. The illustrative examples that we have seen firmly support this perspective.

As we have already stated, we summarize different mechanistic pathways where BP may act as a ferroptosis modulator in Figure 2.

## 5. Concluding Remarks

In summary, BP is packed with diverse ferroptosis modulators, which mainly function as ferroptosis inhibitors. Indeed, ferroptosis inhibition in healthy cells is likely a shared attribute among flavonoids and polyphenols in general, as we have seen. Other intriguing observations, such as ferroptosis selectivity that we have seen for quercetin or contradictory effects of many BP compound examples that we have discussed regarding ferroptosis depending on whether the cell is normal or malignant, warrant further and substantial research efforts. The ferroptosis inhibitory effect of its compounds may endow BP with a considerable ability to mitigate the neurodegeneration process, which is inflicted through decades of alterations and culminates in cell death that, in many NDDs, appears to be driven by ferroptosis. Such interest may also be of great importance in cardiovascular, metabolic, and other age-related diseases. This does not alter its potential to also manage cell death in carcinogenesis due to the wide range of examples that we have seen. In fact, aggressive, metastatic, and treatment-refractory cancer cells are known to be highly sensitive to ferroptosis, and the latter is a striking vulnerability in cancer cells and an acknowledged tumor suppressor with appealing prospects in fighting cancer diseases. Further studies are, therefore, warranted to decipher the real potential of BP in regulating ferroptosis and cell death mechanisms in general. Furthermore, EMT is a newly and highly pursued target to kill cancer cells and promote their sensitivity to ferroptosis. We have already seen that the ability to induce such transition was verified for some BP compounds.

We also underline the necessity to tackle some novel aspects that we have evoked in this work about BP’s potential to modulate ferroptosis. Polysaccharides in BP, and in nature in general, are endowed with many newly unveiled and unattended potentials against aging mechanisms, including oxidative stress and inflammation. The examples that we have seen in BP provide appealing evidence to conduct further studies. On the other hand, phenolamides, which have recently been found to be among the major compounds of BP, remain scarcely studied and should be investigated for their potential in ferroptosis modulation. We have seen numerous examples of very important effects of phenolic acids and polyamines on ferroptosis to decisively incite such investigations.

The multiple encouraging and very promising examples that we have detailed in this paper are added to other diverse effects that we have elucidated in our recent series of publications to firmly bolster the preventive in particular but also the possible curative potential of BP in neurodegeneration and other age-related diseases. However, further focused and phytochemically profiling studies are needed to unveil the real potential of BP in cell death regulation and to understand diverse synergistic, or maybe antagonistic, interactions of its mutual compounds. One of the most formidable translational challenges in our point of view will be to decipher the great dependency on the cellular context to exert cell death regulations by BP compounds, especially for ferroptosis, where a clear-cut distinction between determinant factors to drive the inhibition or induction of ferroptosis is undeniably and fundamentally needed before envisioning any experimental–clinical translation. These determinants will also surely depend on the diversity and mutual complementarity, synergy, or antagonism between diverse compounds in the same BP matrix. Moreover, data regarding the pharmacokinetic and pharmacodynamic profiles of ferroptosis modulators and mechanistic determinants of their intricate and bimodal effects remain poorly understood, and more studies should focus on this point to appraise these important natural activities and use them with sufficient efficiency and safety depending on the type of ferroptosis regulation that we want.
